# Post-discharge Healthcare Usage and Costs From March 2020 Through the Omicron Surge for Individuals Hospitalized With COVID-19

**DOI:** 10.7759/cureus.50663

**Published:** 2023-12-17

**Authors:** Jasmine Leahy, Rebecca Bajracharya, Brian Altonen, Maria Ferreira-Ortiz, Leopolda Silvera, Alfred J Astua

**Affiliations:** 1 Pulmonary Critical Care, Icahn School of Medicine at Mount Sinai, New York, USA; 2 Biostatistics and Epidemiology, Icahn School of Medicine at Mount Sinai, New York, USA; 3 Biostatistics and Epidemiology, Elmhurst Hospital Center, New York, USA; 4 Nursing, Elmhurst Hospital Center, New York, USA; 5 Public Health, Elmhurst Hospital Center, New York, USA; 6 Pulmonary Critical Care, Elmhurst Hospital Center, New York, USA

**Keywords:** primary care, epidemiology and public health, post-covid, long covid, covid-19

## Abstract

Introduction: Despite the rise of post-COVID care centers, few studies exist that quantify the burden of patient healthcare usage and hospital costs after COVID-19 hospitalization. It is essential to target post-COVID follow-up care to the individuals who need it most, such that costs and emergencies are minimized and health and appointment attendance are optimized.

Methods: This was a retrospective cohort comparison among four groups of 50 patients (200 total). Post-discharge healthcare utilization metrics were collected for individuals hospitalized with COVID-19 during the first four surges of the pandemic to compare how patients receive and seek care in the year after they contract COVID-19. A brief cost analysis was done to identify high-usage groups that could be targeted for intervention to decrease post-COVID hospitalization emergencies and burden.

Results: Patients hospitalized during the Omicron surge were scheduled for the most specialist visits on average, significantly higher than average specialist visits in the Delta surge (p<0.05). The Delta surge had significantly less specialty care and missed visits than all other surges (p<0.05) and less primary care than the first two surges of the pandemic (p<0.05). Patients with type 2 diabetes and asthma had the highest overall costs (p<0.05). Females and Hispanic patients had the highest specialty and ED costs (p<0.05).

Conclusion: Each surge reflects a different approach to post-COVID care, with the Omicron surge demonstrating the heaviest usage overall, particularly with specialty visits. Increased specialty referrals may exacerbate rates of missed appointments, while primary care may lower emergency visits. Future approaches to post-COVID care design should identify patients at risk for emergencies and reinstate them with primary care.

## Introduction

The COVID-19 pandemic has defined itself in the United States through distinct waves and surges, whether it be the Delta or Omicron variants [1]. One of the most unique characteristics of COVID-19 has been the emergence of “post-COVID syndrome,” or “long COVID,” which is defined as the series of prolonged symptoms felt by patients starting five weeks to over a year after diagnosis [2, 3]. As many as 10%-35% of people who contract COVID-19 experience post-COVID symptoms, and the number reaches nearly 85% for hospitalized individuals [3]. While several studies have investigated post-COVID syndromes during the initial phases of the pandemic [2, 4, 5], sufficient time has passed to begin observing the long-term effects of COVID-19 on the more recent Omicron and Delta variants.

Studies analyzing earlier pandemic datasets consistently find the most common post- and long COVID symptoms to include fatigue, muscle or joint pain, and shortness of breath [2, 6, 7]. While the vast majority of studies attempt to characterize the symptomology of post-COVID syndromes with clinical sequelae, few have quantified it through the lens of post-diagnosis healthcare utilization metrics, such as the number of specialist referrals and primary care visits [8]. As hospitals begin to open post-COVID care centers, it becomes critical to understand not only how long COVID symptoms are clinically present but also how those symptoms manifest in the number and type of doctor visits. These visits will incur costs for patients, providers, and ultimately healthcare systems, leading to possible insurance mandates and reform. There is an urgent need for this research; streamlining and individualizing post-COVID care will be essential for providers inheriting generations of COVID survivors. As many studies focus on the prevalence of long COVID symptoms in specialty and multidisciplinary clinics [9-11], we must not overlook the importance of primary care as the avenue through which patients seek care for their post-COVID symptoms [12].

Now, years into the pandemic, there is a wealth of data from prior variants that can reveal how patients utilize healthcare differently as the pandemic evolves. To our knowledge, this is the first study that creates a comprehensive comparison of the healthcare burden of post-COVID care during the first four surges of COVID-19, spanning from the initial wave in March 2020 to the January 2022 Omicron wave. We hypothesize that post-COVID healthcare needs will remain high throughout all surges, with heavy costs deriving from specialty and emergency department visits.

This study was conducted at a large public hospital in Queens, NY, one of the most affected areas in the country during the early stages of the pandemic and still a major center for COVID-19 care. Since the probability of having post-COVID symptoms is much greater for hospitalized individuals [3], our study looked at how non-intubated individuals hospitalized with COVID-19 as their primary diagnosis used healthcare services in the 12 months following discharge. We also compared how this utilization changed between the first four surges of the pandemic. Using this information, we estimated what it could potentially cost a single hospital financially to treat post-COVID healthcare needs, with the goal that it could inform other hospital systems on how to best allocate resources for post-COVID care in the future.

Data from this study were presented at the American Thoracic Society Conference 2023 and the Society for General Internal Medicine Conference 2023.

## Materials and methods

Data collection 

We performed a retrospective chart review of de-identified patient files on Epic during each of the first four surges of the pandemic. The surges were categorized as follows based on The Centers for Disease Control and Prevention (CDC) data [1] and internal hospital data: surge #1 (March 2020-May 2020), surge #2 (November 2020-February 2021), surge #3 (August 2021-October 2021), and surge #4 (November 2021-February 2022). This study (approval number: STUDY-22-00736) was determined to be exempt by the IRB Committees at Mount Sinai and Elmhurst Hospital in New York.

We examined the medical and social information of 50 patients for each surge (200 patients total). We focused on non-intubated patients between the ages of 18 and 85 who were hospitalized with COVID-19 as their primary diagnosis at the same large tertiary care center during each of these surges. Intubated patients were excluded from the study due to the increased severity of their illness and other possible confounders that may play a role in their overall morbidity and mortality, which could have presented outliers in the dataset, such as higher appointment numbers and comorbidities. Pregnant patients were excluded given that they may have been using health services or had health conditions that conflicted with our ability to identify appointments and symptoms specifically related to a COVID-19 diagnosis. Patients who died in the hospital or during the first year post-discharge were also excluded, as this could have affected the results by artificially lowering appointment numbers.

We collected the following information for each patient: age, gender, ethnicity, medical history/comorbidities, duration of COVID-19 hospital stay, number and type of specialty visits, primary care visits, and ED visits in the 12 months post-discharge. Comorbidities were collected based on documentation in the medical record. For surges 3 and 4, which represent the Delta and Omicron waves, respectively, we recorded all visits up to the present day of data collection (August 2022). Additionally, we recorded whether an appointment was canceled or the patient did not show up. This way, we determined both the number of “potential” visits (number of scheduled doctor visits) and the number of “actual” visits (number of attended doctor visits). The potential for a visit to take place was considered to incur costs and have a medical impact as a missed appointment prevents the scheduling of another patient in need.

In our analysis, we only included appointments from specialists that have been historically associated with acute COVID-19 and long COVID symptoms, as well as associated routine lab and imaging visits [8, 12]. When possible, we eliminated appointments that were clearly not COVID-related to these specialties. For example, hip and knee imaging were excluded from imaging appointments, and all urology appointments were excluded other than ones pertaining to Foley catheter complications from the COVID-19 hospitalization.

Data analysis

We calculated the financial burden of post-COVID care by multiplying potential visits by the cost per visit. To make this calculation, it was assumed that more than 90% of the patient population was on Medicare or Medicaid. Appointment costs were derived from Healthcare Bluebook data, which is a healthcare price comparison tool reflecting both insurance coverage and physician reimbursement. Costs were calculated by averaging prices within the hospital system itself and from zip codes adjacent to the hospital. The following schema was used: one primary care visit = $164.29, one specialty visit = $214.29, and one emergency room visit = $262.55. Chi-square tests were run to find correlations between the number of patients at different appointment cost levels. This was further confirmed with linear-by-linear association and Fisher’s exact tests. Paired t-tests were run comparing average appointment numbers between surges. A p-value of less than 0.05 was considered significant.

## Results

Demographics

**Figure 1:**
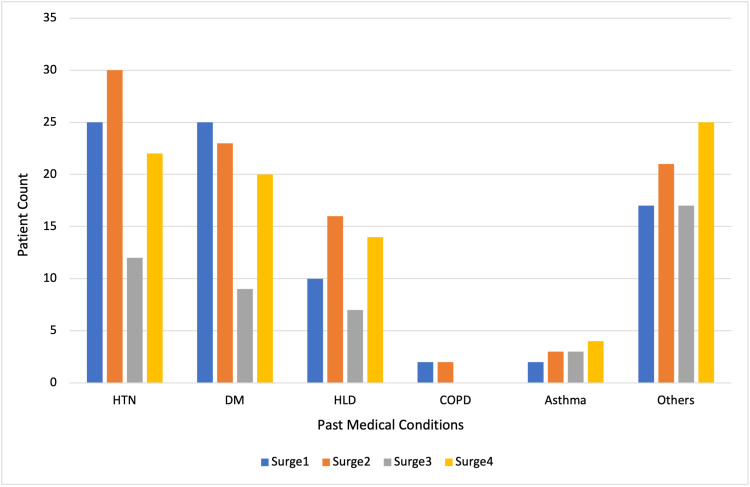
Distribution of past medical conditions by surge HTN: hypertension; DM: diabetes mellitus; HLD: hyperlipidemia; COPD: chronic obstructive pulmonary disease

Overall, there were 129 male and 71 female patients, with an average age of 57 years. The average BMI was 29 kg/m^2^, and the average length of hospital stay was eight days. Notably, 57.5% (115/200) of patients were Hispanic or Latino, consistent with the hospital’s typical patient population. Of the study group, 6% were Black patients, 4.5% were White patients, 15.5% were Asian patients, and 16.5% were considered “other” due to a lack of documented race. Hypertension was the most common underlying medical condition, followed by type 2 diabetes (Figure 1).

Surge 3 had the overall lowest prevalence of comorbidities (Figure 1) and the youngest average age (Table 1).

**Table 1:** Demographics overview broken down by surge

Metric	Subgroup	Patient count	Average value	Surge 1 average	Surge 2 average	Surge 3 average	Surge 4 average
Age (years)	All	200	56.6	57	60	48	61
By gender	Male	129	53.3	57	57	46	56
--	Female	71	62.1	58	64	54	69
By race/ethnicity	Hispanic	115	55.7	54	61	48	59
--	Black	12	49.8	80	62	44	N/A
--	White	9	54.0	67	N/A	51	46
--	Asian	23	57.3	63	53	49	N/A
BMI (kg/m^2^)	All	200	29.03	29.76	28.55	30.72	27.34
By gender	Male	129	28.40	28.18	27.38	30.97	26.52
--	Female	71	30.17	31.82	30.81	29.97	28.38
By age group (years)	20-29	9	34.62	37.90	28.18	36.54	26.29
--	30-39	23	30.31	24.30	34.24	32.69	27.10
--	40-49	32	28.74	32.05	28.30	29.56	25.83
--	50-59	47	28.18	29.37	27.02	28.08	29.01
--	60-69	43	30.25	31.83	28.26	30.03	29.69
--	70-79	28	25.98	25.86	26.98	23.36	26.17
--	80+	18	28.86	28.41	30.50	32.78	24.93
By race/ethnicity	Hispanic	115	29.60	31.11	29.04	31.22	27.32
--	Black	12	32.43	34.94	24.86	34.01	N/A
--	White	9	27.33	33.89	N/A	25.32	26.22
--	Asian	23	27.13	26.42	28.88	25.07	N/A

Healthcare usage by surge 

Overall, 64.5% (129/200) of patients sought or were referred to primary care at least once in the year after discharge. Fifty-eight percent (116/200) of patients were scheduled for specialty care. There were 36 patients (18%) with at least one ED visit. Eighty-six percent (31/36) of ED patients had at least one missed appointment, compared to 58% (95/164) of patients with no ED visits (p=0.002). 

**Figure 2:**
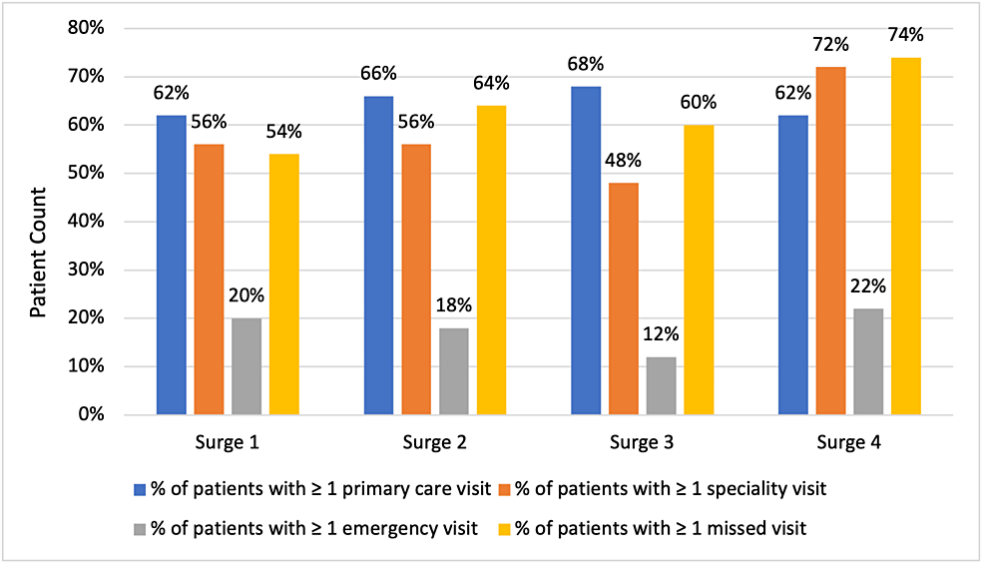
Patient healthcare utilization is defined as the percentage of patients with at least one primary care visit, specialty visit, ED visit, or missed visit.

Figure 2 presents a breakdown of appointment type by surge.

In surge 1, 62% (n=31) of patients were scheduled for primary care, 56% (n=28) were scheduled for specialists, 20% (n=10) went to the ED at least once, and 54% (n=27) of patients missed at least one appointment. Surge 1 had the highest average primary care appointments per patient (n=3.8), significantly more than surges 3 and 4 (Table 2).

**Table 2:** Average number of primary care, specialty, and missed appointments up to 12 months after COVID-19 hospital discharge by surge.

Surge #	1	2	3	4
Average primary care visits at 12 months	3.8	3.44	1.84	1.96
Average specialty visits at 12 months	4.92	6.48	2.98	6.94
Average missed visits at 12 months	2.8	3.64	1.7	3.2

In surge 2, 66% (n=33) of patients were scheduled for primary care, 56% (n=28) were scheduled for specialists, 18% (n=9) visited the ED, and 64% (n=32) missed at least one appointment. Surge 2 had the highest average missed appointments per patient (n=3.64), significantly greater than surge 3 (Table 3).

**Table 3:** Difference between pairs of surges for primary care, specialty, and missed visits up to 12 months after COVID-19 hospital discharge.

Surge pairs	1 and 2	1 and 3	1 and 4	2 and 3	2 and 4	3 and 4
Average primary care visits at 12 months	No significant difference	1>3 (p=0.001)	1>4 (p=0.001)	2>3 (p=0.0005)	2>4 (p=0.0005)	no sig. difference
Average specialty visits at 12 months	No significant difference	1>3 (p<0.05)	No significant difference	2>3 (p<0.05)	No significant difference	4>3 (p=0.006)
Average missed visits at 12 months	No significant difference	1>3 (p=0.005)	No significant difference	2>3 (p=0.002)	No significant difference	4>3 (p<0.05)

In surge 3, 68% (n=34) of patients were scheduled for primary care, 48% (n=24) were scheduled for specialists, 12% (n=6) went to the ED, and 60% (n=30) missed at least one appointment. Notably, surge 3 had the lowest average primary care, specialty, and missed appointments per patient of all four surges (Tables 2-3).

For surge 4, 62% (n=31) were scheduled for primary care, 72% (n=36) were scheduled for specialists, 22% (n=11) went to the ED, and 74% (n=37) missed at least one appointment. Patients in surge 4 were scheduled for the most specialist visits on average (n=7). This was significantly higher than average specialist visits in surge 3 (p<0.05; Tables 1-2). Surge 4 patients were also scheduled for significantly fewer primary care appointments than patients in surges 1 and 2 (p<0.05; Tables 2-3).

Cost analysis

The estimated cost of post-COVID primary care was $455.40/person (2.76 average visits * $165/appointment). Specialty visits totaled $1,142.17/person (5.33 average visits * $214.29/appointment). For the 36 patients who visited the ED, the average cost of ED visits per person was $716.76 (2.73 average visits * $262.55). Extrapolating from Tables 2-3, surge 3 had the lowest primary care (p<0.05), specialty, and ED costs per patient.

**Figure 3:**
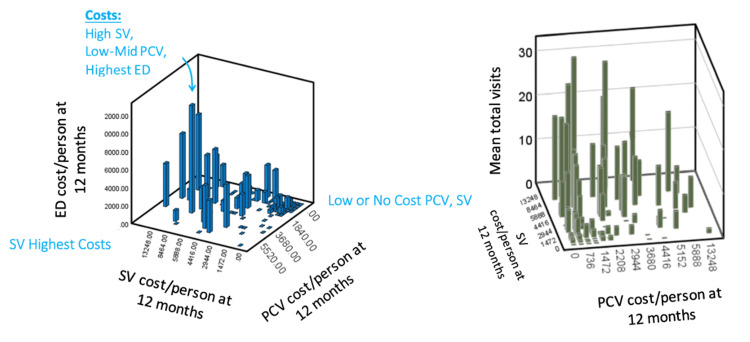
Left: A three-dimensional (3D) graph of specialty visit costs (SV), primary care visit costs (PCV), and ED costs in dollars. Right: A 3D graph of SV costs, PCV costs, and mean total visits

Overall, patients with a combination of low primary care costs and high specialty visit costs had the highest ED costs and highest average appointments (Figure 3).

Additionally, patients with type 2 diabetes and asthma had higher overall costs than patients without those conditions (p<0.05). Females had higher specialty and ED costs than males (p<0.05). Specialty and ED costs were also higher for Hispanic patients when compared to non-Hispanic patients (p<0.05). Age was not a significant predictor of cost.

## Discussion

Healthcare utilization across surges

Patients sought post-COVID healthcare differently depending on the surge in which they were hospitalized, representing a potential change in physician and/or patient attitudes towards post-COVID healthcare. Notably, specialists were sought out more than primary care, especially in the more recent surge 4, which corresponds to the Omicron outbreak in the U.S. Despite the high rates of specialty appointments made, emergency and missed visits were frequent throughout all surges. Surge 4, which corresponds to the Omicron variant in the U.S., was notable for the most specialty, emergency, and no-show/canceled visits (Tables 1-2). This is particularly outstanding considering only six months had passed after surge 4 when the data was collected, whereas surges 1 and 2 contained a complete 12-month period. One can assume that the gap in healthcare usage between surge 4 and previous surges will only grow larger with time. Surge 3, representative of the U.S. Delta variant, had a comparatively healthier and younger patient population compared to other surges, which could explain the low rates of healthcare usage (Figure 1). This is consistent with patterns observed in the Delta variant affecting younger patients with fewer comorbidities [13].

The increased healthcare engagement seen in surge 4 (Figure 2; Table 2-3) may be due to a variety of reasons. One possibility is that post-COVID symptoms may be worsening with successive variants. Though the Omicron variant appears to have a milder acute presentation [14], that does not preclude it from causing long-term symptoms. The high rates of reinfection during the Omicron surge may also contribute to increased healthcare usage. A recent study showed that COVID-19 reinfections increased a patient’s six-month probability of death, hospitalization, and clinical symptoms [15]. More research is needed as the pandemic continues to track the evolution and risk factors of long COVID.

Another possible explanation for increased healthcare usage in the Omicron surge is that both physicians and patients are becoming increasingly attuned to the possibility of long COVID and its presenting symptoms, resulting in more referrals and/or scheduled appointments. As more long COVID research is published, it often appears as if any symptom could be considered a post-COVID sequelae. While symptoms like shortness of breath, fatigue, and long-term anosmia and ageusia are well-documented long COVID presentations with physiological explanations, other proposed long COVID symptoms such as tinnitus, hearing loss, and insomnia lack robust biological explanations [16-18]. 

The role of primary care

Submitting numerous specialty referrals has been proposed as a model for post-COVID hospitalization care, given the multi-organ nature of the disease [8, 19]. However, surge 4 saw the highest rate of missed appointments in the pandemic despite also having the most scheduled appointments (Figure 2). Furthermore, our cost analysis showed that patients who accrued high specialty and low primary care costs visited the ED most, possibly making the current post-COVID multidisciplinary model of care less cost-effective than previously thought. Increased specialty referrals may also exacerbate rates of no-show appointments.

Primary care appointments may be protective against post-COVID ED visits, decreasing mean annual appointments and thus overall costs (Figure 3). A prior non-COVID study showed that patients who saw comprehensive primary care physicians without specialty referral had fewer ED visits than patients who saw both primary care and specialists [20]. A later study replicated the finding that primary care offices offering a wide range of services were associated with less ED utilization and lower Medicare costs [21]. Improved inter-physician communication may improve continuity of primary care and, thus, emergency admissions [22].

Based on the results of our study, post-COVID care may benefit from this model of broad primary care and well-connected provider networks. This could include the concept of an ED-designated COVID navigator (CN) who would ensure that patients arriving at the ED with a history of COVID-19 hospitalization could be quickly connected to primary care to prevent emergency revisits for similar complaints. The CN could embody a physical person; more likely, however, the CN would be a systems process in the hospital’s electronic medical record (EMR) that could act as an expedited referral process. Though we conducted this study at a hospital with a majority of Medicaid and Medicare patients, this model could be widely applicable to hospitals with any combination of payer distributions due to the common shared goal of lowering costs and optimizing health outcomes and appointment attendance.

Patient demographic patterns

It is critical to acknowledge that our study centered on a majority non-White, publicly insured patient population. Non-White, male patients tend to seek post-COVID care at lesser rates despite a more severe illness [8, 19]. Additionally, patients with a lower socioeconomic background are more likely to miss appointments [23]. This was reflected in our study, which demonstrated high overall rates of no-show appointments and ED visits. In particular, Hispanic patients accrued significantly larger amounts of hospital costs than other groups. It is therefore imperative that future post-COVID hospitalization initiatives focus on underserved patients.

Limitations

Our study was intended to provide a starting point for calculating the cost of post-COVID healthcare needs, which is immensely complicated. In reality, the cost of a single appointment will vary greatly depending on a patient’s needs and comorbidities. Healthcare Bluebook data is also limited by the variation in pricing across providers and institutions. Future studies should add more complexity to this initial cost analysis. Another limitation was the inability to perfectly separate COVID-related appointments from non-COVID appointments, despite best efforts. It is possible that appointments made outside of the hospital’s network were not visible on the electronic medical record; however, these are likely few in number.

The patient population is representative of a single community and may not reflect that of the entire U.S. Our exclusion criteria may have resulted in selection bias. Further research should consider expanding the population of interest, particularly to rural areas, which may face unique barriers to care. Additionally, this study was only among non-intubated, hospitalized patients. Although only 5%-20% of COVID-19 inpatients are intubated at a given moment, according to the CDC, it is possible that excluding this population may affect the generalizability of the data to the overall hospitalized COVID population [24]. Furthermore, up to two-thirds of non-hospitalized COVID patients have been shown to utilize healthcare in the 180 days after infection [8] and even self-report worse post-COVID health than hospitalized patients [19], making them an important subgroup to consider for future studies.

## Conclusions

The pandemic and its long-term health ramifications continue to evolve with each variant encountered, with the more recent Omicron variant showing an increase in healthcare usage. The importance of primary care may be currently overlooked in the context of post-COVID care, given that patients who used less primary care had higher rates of emergency visits. Not only is it important to research post-COVID symptoms, but it is also critical to study how patient behavior changes over time, which may give early indications of the progression of the disease. Vaccination status, current circulating COVID levels, age, and comorbidities are all examples of changing variables that will continue to shape how patients seek care after COVID-19 infection. There is a need to actively reconnect post-COVID hospitalization patients with primary care in order to lower specialty costs, missed appointments, and ED visits. On an institutional level, this reemphasizes the need for increased investment in preventative medicine and cross-disciplinary provider communication. Future research could include the development of a COVID-19 patient navigator/liaison, as mentioned, to reinstate patients with care that is not only cost-effective but most beneficial for their health and needs.
